# Clinical benefits of oral anticoagulants in atrial fibrillation patients with dementia: a systematic review and meta-analysis

**DOI:** 10.3389/fcvm.2023.1265331

**Published:** 2023-09-05

**Authors:** Dayang Wang, Xiaoqing Xu, Xiaowan Han, Jing Xie, Hufang Zhou, Wenhua Peng, Guozhong Pan

**Affiliations:** ^1^ Cardiovascular Institute, Dongzhimen Hospital, Beijing University of Chinese Medicine, Beijing, China; ^2^ Second Department of Cardiology, Dongzhimen Hospital, Beijing University of Chinese Medicine, Beijing, China; ^3^ Department of Endocrinology, Beijing Hepingli Hospital, Beijing, China

**Keywords:** atrial fibrillation, dementia, oral anticoagulants, all-cause mortality, meta-analysis

## Abstract

**Background:**

The management of atrial fibrillation (AF) with oral anticoagulants (OAC) is generally recommended to reduce the risk of stroke. However, the decision to prescribe these medications for patients with AF and dementia remains controversial.

**Methods:**

A systematic review and meta-analysis of retrospective cohort studies were conducted. The search encompassed PubMed, Cochrane Library, Web of Science, and Embase databases from inception until May 1st, 2023, with language limited to English. Eligible studies included comparisons between exposure to OAC vs. non-OAC in the AF population with dementia or cognitive impairment. Studies that compared the effects of direct oral anticoagulants (DOAC) and vitamin-K antagonists were also included. The primary outcome was all-cause mortality, and the secondary outcomes were ischemic stroke and major bleeding. This study was registered with PROSPERO (No. CRD42023420678).

**Results:**

A total of five studies (*N* = 21,962 patients) met the eligibility criteria and were included in this review. The follow-up duration ranged from 1 to 4 years. Meta-analysis demonstrated that OAC treatment was associated with a lower risk of all-cause mortality in AF patients with dementia with a hazard ratio (HR) of 0.79 and a 95% confidence interval (CI) ranging from 0.68 to 0.92, compared to non-OAC treatment. No statistical differences were observed in the risk of major bleeding (HR = 1.12, 95% CI: 0.88–1.42) or ischemic stroke (HR = 0.77, 95% CI: 0.58–1.00). Three studies reported comparisons between DOAC and warfarin; however, pooled analysis was not performed due to heterogeneity.

**Conclusion:**

The use of OACs in individuals diagnosed with both AF and dementia holds the potential to reduce all-cause mortality rates, thereby improving the overall clinical prognosis within this specific population.

**Systematic Review Registration:**

https://www.crd.york.ac.uk/prospero/display_record.php?ID=CRD42023420678, PROSPERO identifier, CRD42023420678.

## 1. Introduction

Ischemic stroke is the primary risk contributing to an unfavorable prognosis in patients diagnosed with atrial fibrillation (AF) (1). Oral anticoagulant (OAC) therapy plays a pivotal role in preventing ischemic stroke in AF patients, significantly improving clinical outcomes, reducing overall mortality, and lowering stroke incidence (2). Despite the association of OAC use with an increased risk of bleeding, the overall benefits outweigh the risks of not using OACs (2).

However, controversy surrounds the benefits of anticoagulant therapy in individuals with pre-existing dementia or cognitive impairment. Good adherence to OACs is foundational for clinical benefits (3). However, individuals with dementia exhibit significantly lower medication adherence, even with medical care, compared to those without dementia (4), significantly impacting the ischemic protection benefits of OACs (5). On the other hand, the risk of intracranial hemorrhage associated with OAC use is also increased in individuals with dementia (6). Furthermore, individuals with dementia are generally older, have multiple comorbidities, and have a shorter life expectancy. Therefore, further exploration is necessary to determine whether individuals with dementia can still derive benefits from OAC therapy.

Current studies have yielded divergent conclusions regarding the impact of OAC therapy on individuals with dementia. Some studies suggest that OACs can improve prognosis in patients with dementia (7, 8), while others indicate that it may worsen outcomes (9). On the other hand, direct oral anticoagulants (DOACs) have lower adherence requirements compared to vitamin K antagonists (VKA), or warfarin, as they do not require regular monitoring of international normalized ratio (INR). Theoretically, the use of DOACs in patients with non-valvular atrial fibrillation may offer greater benefits in this population compared to VKAs.

Therefore, we conducted a systematic review and meta-analysis aiming to evaluate the benefits of OAC use in patients diagnosed with AF and dementia/cognitive impairment, as well as to determine whether DOACs offer greater advantages compared to VKAs.

## 2. Methods

The protocol of this studies was registered with PROSPERO (No. CRD42023420678) (10). This review was planned, conducted, and reported according to the Preferred Reporting Items for a Systematic Review and Meta-analysis 2020 update (PRISMA 2020) (11); the PRISMA checklist was attached in Supplementary Appendix.

### 2.1. Data sources and searches

The searches were conducted in four databases: PubMed, EMBASE, Cochrane Library, and Web of Science, covering publications from the inception until May 1st, 2023. To perform the search, we utilized subject terms and free words, including “dementia,” “Alzheimer’s,” “cognitive impairment,” “anticoagulation,” “direct oral anticoagulants,” “antithrombins,” and others. Before final analyses, additional searches were performed, and relevant studies were retrieved for inclusion. For detailed search strategies, refer to Supplementary Material.

### 2.2. Eligibility criteria and study selection

Studies were considered eligible for inclusion if they met following criterion: (1) study type: cohort study; (2) patients: diagnosed with AF and dementia/cognitive impairment at baseline; (3) exposure: receiving OAC; control: non-OAC; (4) outcome: estimated the risk for all-cause mortality, ischemic stroke and major bleeding with adjusted hazard ratio (HR) and 95% confidence interval (95% CI); The process of study selection advanced as follows: (1) exclude duplicate publications; (2) read study titles and abstracts and exclude the non-cohort studies; (3) read the abstracts and exclude studies not related to AF; (4) read the full-text and exclude the studies with non-conforming exposures, populations, and outcomes. Two independent reviewers (DW and XX) scanned titles and abstracts according to the inclusion criteria. Any discrepancy regarding searches and selection was discussed in consultation with and resolved by a third reviewer (GP). If a study potentially met the inclusion criteria, the full text was retrieved for further inspection.

### 2.3. Data extraction

Upon identifying the included studies, two reviewers independently conducted data extraction. The extracted data encompassed the following categories: (1) general information, including study name, year of publication, country of study, population, sample size, follow-up duration, reported outcomes, type of anticoagulation, and dementia ascertainment; (2) baseline information, comprising sex, age, percentage of diabetes, hypertension, prior ischemic stroke, heart failure, CHA2DS2-VASc score, and estimated glomerular filtration rate (eGFR); (3) adjusted HR and 95% CI for outcomes; (4) adjusted confounders of HR. After extraction, the data were thoroughly checked. Any discrepancies were verified and resolved by a third reviewer (GP). Records of studies were managed with the Endnote X9 software (RRID:SCR_014001). In this review, the management of missing values followed the processing method reported in the original studies.

### 2.4. Outcomes and definitions

The primary outcome in this study is all-cause mortality. The secondary outcome is ischemic stroke and major bleeding. The ascertainment strategy of dementia or cognitive impairment include but are not restricted to Reisberg Global Deterioration Scale (RGDS), Cognitive Performance Scale (CPS); Montreal Cognitive Assessment (MoCA), International Classification of Diseases (ICD) code, and database documented records. OAC include DOAC or VKA. Antiplatelet drugs (aspirin for example) are not involved.

### 2.5. Risk of bias and sensitivity assessment

The risk of bias in cohort studies was assessed by two reviewers (DY and XX) independently using the Newcastle-Ottawa Scale (NOS) with a scoring range from 0 to 9 (12). Regarding the follow-up time in the NOS, due to the relatively high incidence of outcomes in the very elderly population, we stipulated that a minimum follow-up of 6 months would be scored to account for the occurrence of primary and secondary outcomes. Potential publication bias was evaluated through funnel plots generated by RevMan 5.4.1 software (RRID: SCR_003581) (13).

Sensitivity analysis was systematically performed by sequentially excluding individual studies from the analysis to examine their impact on the results.

### 2.6. Statistical analysis

Meta-analyses were conducted for comparable studies. Primary and secondary outcome effect measures with adjusted HR and 95% CI were pooled using RevMan 5.4.1 software. The value of logHR and its standard error (SE) required for pooled analysis were derived from the HR and 95% CI as reported in the original articles. Results are presented visually using forest plots. Heterogeneity was quantitatively assessed using Higgins's index (*I*^2^), and *p* value. Random-effect models were applied independently of the levels of *I*^2^ due to the inter-study heterogeneity derived from population OAC type, follow-up course etc.; In cases where quantitative data were insufficient or highly heterogeneous, a descriptive synthesis approach was employed. All statistical tests were two-sided and set at a significance threshold of *p* < 0.05.

## 3. Results

### 3.1. Study selection

A total of 2,501 records were obtained from the initial search, and 1,032 duplicate records were removed.

**Figure 1:**
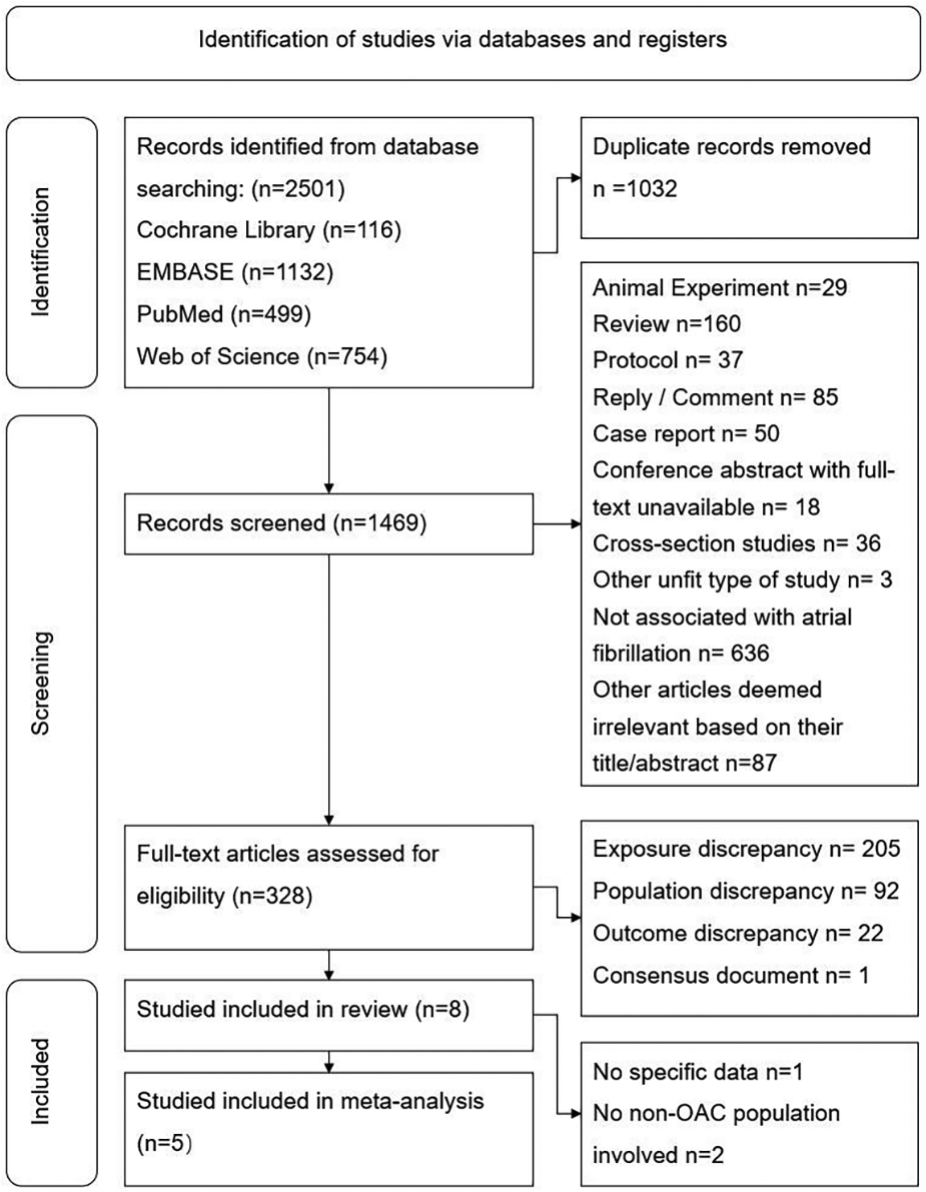
Flow chart of the article selection procedure for meta-analysis.

We screened the remaining 1,469 records by reading their titles and abstracts and excluded 1,141 records as they were deemed inappropriate article types or lacked association with AF. After assessing the full text of the remaining 328 articles, we excluded 207 records due to discrepancies in exposure, 92 due to discrepancies in the study population, and 22 due to discrepancies in outcomes. One article was identified as a consensus document and was not included in the systematic review. Ultimately, eight studies met the inclusion criteria and were included in the systematic review. Among them, one study [Madhavan et al. (14)] failed to provide detailed raw data of HR or 95% CI; two studies only reported comparison between DOAC and VKA (15, 16). Consequently, five studies were included in the meta-analysis (7–9, 17, 18). The process of study selection is visually depicted in Figure 1.

### 3.2. Study characteristics

**Figure 2:**
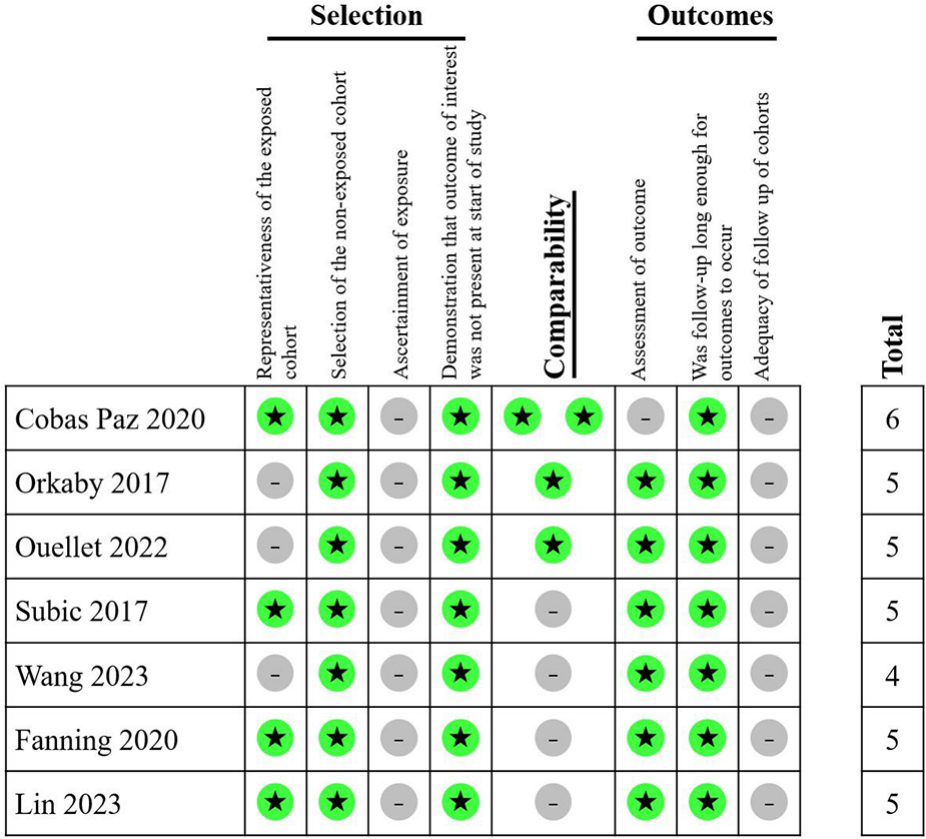
Newcastle-Ottawa Scale for included studies in meta-analysis.

A total of 21,962 patients with dementia and AF from five studies were included in meta-analysis. All five studies were retrospective cohort studies. The follow-up course ranged from 1 to 4 years. All five studies provided outcomes of all-cause mortality, major bleeding, and ischemic stroke. In terms of type of anticoagulants, three studies involved VKA or DOAC, while two studies involved only VKA. The methods of dementia ascertainment varied from the studies. RGDS, CPS and MoCA were employed in one study respectively. Two studies used ICD code in the previous database. The NOS score ranged from 4 to 6 in included studies (Figure 2). General characteristics of the studies are shown in Table 1. The average age of patients included in all five studies exceeded 75 years old, with a high prevalence of concomitant diseases. Table 2 displays the baseline characteristics of the studies. Significant differences in the rate of concomitant diseases were observed between the OAC and non-OAC groups. All five studies utilized multivariable model adjustment to control for HR. Supplementary Table S1 lists the adjustment of confounders in the included studies.

**Table 1:** General characteristics of the included studies.

Study ID	Country	Population	Sample Size	Follow-up Course (year)	Anticoagulants	Dementia ascertainment	Study Type
Cobas Paz et al. (18)	Spain	Common population	221	2.8	VKA/DOAC vs. OAC withdrawal	RGDS (5–7)	Retrospective
Orkaby et al. (8)	U.S.	Veterans	1215	4.0	VKA vs. OAC withdrawal	ICD-9 code	Retrospective
Ouellet 2022 (17)	U.S.	Nursing home residents	14,877	1.0	VKA/DOAC vs. OAC withdrawal	CPS (5–6)	Retrospective
Subic et al. (7)	Sweden	Common population	5121	1.7	VKA vs. OAC withdrawal	ICD-10 code	Retrospective
Wang et al. 2023 (9)	U.S.	Patients with contraindications to anticoagulation were excluded	528	2.0	VKA/DOAC vs. OAC withdrawal	MoCA (<23)	Retrospective
Fanning et al. (16)	U.K.	Common population	2,399	0.8	DOAC vs. VKA	Not mentioned	Retrospective
Lin et al. (15)	U.S.	Common population	40,350	0.5	DOAC vs. VKA	ICD-9/10 code	Retrospective

VKA, Vitamin K antagonist; DOAC, direct oral anticoagulants; RGDS, Reisberg Global Deterioration Scale; CPS, Cognitive Performance Scale; ICD, International Classification of Diseases; MoCA, Montreal Cognitive Assessment.

**Table 2:** Baseline characteristics of the included studies.

Study ID	Age, years	Sex (female), %	Diabetes, %	Hypertension, %	Ischemic stroke, %	Heart failure, %	CHA2DS2-VASc score	eGFR, ml/min/1.73 m2
Cobas Paz et al. (18)	89.4	69.7	18.1	65.6	18.1	18.1	4.3 ± 1.3	59.2 ± 18.6
Orkaby et al. (8)	79.5	1.4	45.2	95.0	25.5	59.2	–	≥60 (56.4%) 30–59 (39.9%) <30 (9.7%)
Ouellet 2022	≥80 (82.7%)	72.0	38.0	89.7	55.2	42.5	6.19 ± 1.58	–
Subic et al. (7)	81.7	47.2	19.4	62.8	22.7	37.2	–	–
Wang et al. 2023	75.5	49.9	27.8	90.9	9.8	37.2	4.43	70.5
Fanning et al. (16)	82	54	22.2	68.3	27.3	13.6	4.0 (3.0–5.0)	–
Lin et al. (15)	82.84	59.5	42.9	92.2	42.5	83.5	5.79 ± 1.60	–

“–”, baseline data was not mentioned in the original articles.

eGFR, estimated glomerular filtration rate.

### 3.3. Total results of meta-analysis

#### 3.3.1. OAC vs. non-OAC

**Figure 3:**
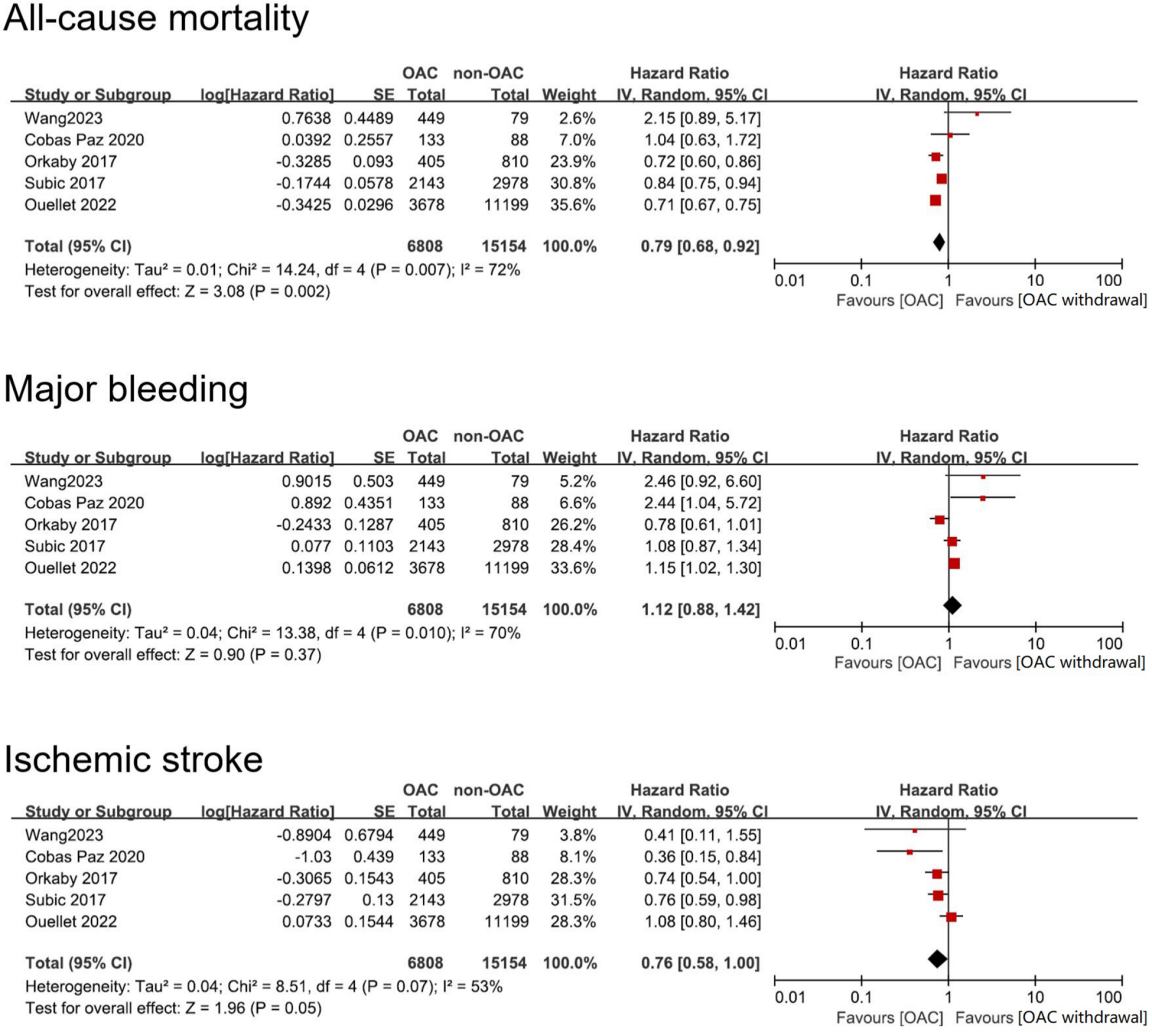
Forest plot of meta-analysis of studies involving efficacy and safety results between OAC and non-OAC in AF population with dementia. SE, standard error.

All five studies reported the outcome of all-cause mortality. The heterogeneity analysis yielded an *I*^2 ^= 72% with a *p* value of 0.007. Consequently, the random-effects model was adopted for the meta-analysis, resulting in a pooled HR of 0.79 (95% CI: 0.68–0.92), indicating a lower risk of all-cause mortality in AF patients with dementia treated with OAC compared to non-OAC (Figure 3).

All five studies reported outcome of major bleeding. Heterogeneity analysis showed a result of *I*^2 ^= 70% with a *p* value of 0.01. Therefore, we adopted the random-effects model to perform the meta-analysis and reached a pooled HR = 1.12 (95% CI: 0.88–1.42). The data indicated that OAC use and non-OAC use manifested no statistical difference in terms of risk of major bleeding (Figure 3).

Similarly, all five studies reported the outcome of ischemic stroke. Heterogeneity analysis showed a result of *I*^2 ^= 53% with a *p* value of 0.08. Therefore, we adopted the random-effects model. The meta-analysis reached a pooled HR = 0.77 (95% CI: 0.58–1.00), which showed that OAC treatment had no benefit in lowering risk of ischemic stroke (Figure 3).

#### 3.3.2. DOAC vs. VKA

**Figure 4:**
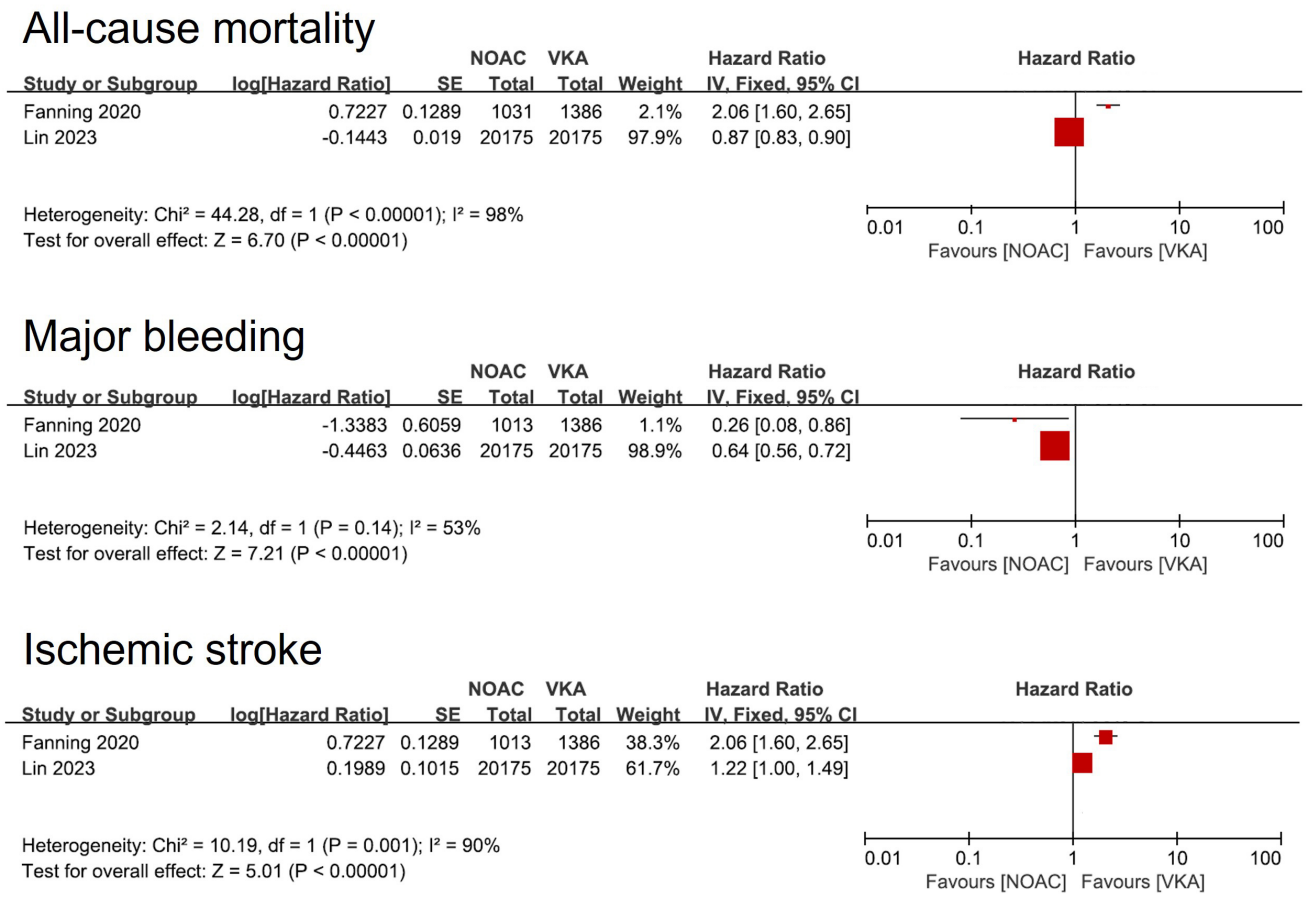
Forest plot of meta-analysis of studies involving efficacy and safety results between DOAC vs. VKA. SE, standard error. DOAC, direct oral anticoagulants. VKA, Vitamin K Antagonist.

Three studies compared clinical outcomes between DOAC and VKA [Lin et al. (15); Fanning et al. (16) and Wang et al. (9)]. Due to the heterogeneity of the three studies (including variations in population, DOAC type, and follow-up time, etc.), the pooled analysis was not conducted, and only the individual study outcomes were described. In the first two studies, the warfarin group had a higher risk of major bleeding and a lower risk of Ischemic stroke than DOAC group. In terms of all-cause mortality, there were differences between the two studies (Figure 4). Furthermore, Wang et al. (9) compared the benefits of a composite outcome involving major bleeding or all-cause mortality, which did not show any superiority (HR = 1.17, 95% CI: 0.78–1.76).

### 3.4. Sensitivity analysis

In the sensitivity analysis, we sequentially removed each study from the pooled analysis to investigate its influence on the overall effect size. By omitting study Ouellet, 2022 and Orkaby, 2017, the pooled effect of all-cause mortality HR changed to 0.86 (95% CI: 0.69–1.06) and 0.83 (95% CI: 0.68–1.02) respectively. The effect on ischemic stroke was similarly changed by omitting study Ouellet 2022 (HR = 0.70, 95% CI: 0.57–0.87). The pooled effects of major bleeding remained unchanged with the omission of a single study (Supplementary Figure S1).

### 3.5. Publication bias

The funnel plot (Supplementary Figure S2) of OAC treatment on all-cause mortality, major bleeding and ischemic stroke showed asymmetry, indicating possible publication bias.

## 4. Discussion

### 4.1. Main findings and interpretations

Our study revealed that AF patients with concomitant dementia can derive benefits from OAC therapy. The meta-analysis demonstrated a reduction in all-cause mortality with OAC therapy, and this effect may not be solely explained by its protective effect against ischemic stroke. There was no significant increase in the risk of major bleeding.

Presently, the HAS-BLED score is recommended for stratifying bleeding risk in anticoagulant therapy in AF patients. Despite dementia being categorized as a high-risk factor for bleeding in the guidelines (19), there is currently insufficient evidence to support discontinuing OAC in this population. Our findings suggest that AF patients with concomitant dementia can still benefit from OAC therapy, consistent with the prevailing guideline recommendations.

The five cohort studies showed heterogeneity in the three outcomes of interest. In relation to all-cause mortality, three large-sample studies demonstrate that OAC is superior to non-OAC. The results of these three studies confirm the beneficial effect of OAC in reducing all-cause mortality in the pooled analysis. Two studies indicated that OAC has a higher bleeding risk than non-OAC. One study (Orkaby 2017) exhibited a trend inconsistent with the overall results, suggesting a tendency towards reduced bleeding risk with OAC compared to non-OAC. The extended follow-up duration in Orkaby 2017 study led to a relatively high incidence of major bleeding, significantly impacting the overall results. Nevertheless, sensitivity analysis revealed that excluding this study did not impact the overall results of the study. Three studies demonstrated that the stroke risk with OAC is lower than with non-OAC, but the overall results did not indicate a significant benefit. The heterogeneity mainly originated from the large-sample study Ouellet 2022, and sensitivity analysis indicated that excluding this study would impact the stability of the results (overall effect *p* = 0.001). The inclusion of the five studies resulted in high heterogeneity concerning the study population and outcomes (*I*^2^ reaching 72%, 70%, and 53% respectively). Nevertheless, the five studies employed adjusted confounders that generally accounted for known factors that may cause bias, and we believe that including these five studies in the pooled analysis is appropriate.

Regarding the comparison between DOACs and VKAs, theoretically, the reduced adherence requirement makes DOACs more suitable for patients with concurrent dementia, potentially leading to improved outcomes. However, only three articles meeting the inclusion criteria were retrieved, and they exhibited significant heterogeneity in the results. Additionally, one of the articles solely provided data on composite outcomes. Therefore, the results of this study are not sufficient to draw a conclusive recommendation favoring DOACs.

### 4.2. How can OAC benefit patients with AF and dementia?

This study showed that OAC reduced all-cause mortality, but did not demonstrate a preventive effect on ischemic stroke in AF patients with concurrent dementia. The discrepancy can be attributed to benefits of OAC in other comorbidities. Elderly individuals commonly experience comorbid hypercoagulability, which subsequently elevates their risk of thrombotic events (20). The utilization of OAC may lead to a reduction in these adverse events. For instance, elderly individuals with dementia often experience bedridden conditions, which consequently increases the risk of venous thrombosis. The advantage of employing OAC in this particular population might be attributed to a decrease in pulmonary embolism. Additionally, among the five studies included, there was a significant prevalence of concomitant coronary artery disease. Drawing from the established benefits of OAC in atherosclerotic cardiovascular disease (21), we speculated that the patients could also derive benefits from a decrease in acute cardiovascular events. These potential advantages may account for the observed decline in all-cause mortality among the patients.

However, the clinical benefits for individuals with both AF and dementia present a complex and challenging issue. Quality of life remains a significant aspect of clinical benefits even for patients with concurrent dementia. While existing evidence suggests that administering OAC to reduce stroke incidence can increase quality-adjusted life-years at high health economics cost (22), there is a lack of direct evidence demonstrating an improvement in the quality of life in this population. Apart from the decline in quality of life caused by complications such as stroke or major bleeding, the treatment itself (particularly strict adherence to medical advice, including the use of VKA and monitoring the INR) often imposes significant burdens on both patients and care providers. As a result, quantifying the quality of life for this population remains a challenging endeavor. The only quantifiable benefits for such patients may lie in the extension of life and the reduction in mortality rates.

### 4.3. Relationship with previous studies

Stratified management is critical for anticoagulants treatment of AF. Currently, the CHA2DS2-VASc and HAS-BLED scores are the recommended tools for risk stratification in guidelines. However, clinical situations are complex, making the decision to use OAC in high-risk patients quite difficult. Previous studies have identified factors that lead to discontinuation of OAC, including advanced age (23), concomitant chronic kidney disease (24, 25), concomitant chronic obstructive pulmonary disease (24), and dementia/cognitive impairment (4, 26). The main reasons for OAC discontinuation in dementia patients are twofold: on one hand, ensuring adherence in these patients is challenging, and non-adherence or overdosing is common, leading to reduced benefits (27); on the other hand, doctors or patients may be concerned about an increased risk of major bleeding (28). However, discontinuing OAC based on these concerns lacks sufficient evidence. Therefore, previous viewpoints have suggested that dementia is not reason to withhold anticoagulation in AF management (29). The results of our study support this viewpoint.

### 4.4. Limitations

Our study has several limitations: Firstly, All the included studies are retrospective cohort studies. The issue of baseline imbalance is inevitable. Although multivariate adjustment can be performed, it cannot completely eliminate bias caused by baseline differences. However, conducting randomized controlled trials in this population is extremely challenging due to issues of adherence control and ethical considerations. Therefore, there are currently no RCTs available in this regard. Secondly, overall medication adherence data were not obtained. These data are assumed as a premise, but very few of the included studies provided this data. Acquiring such data is challenging. Therefore, it was not included in the analysis. Next, there was significant heterogeneity among included studies. The included studies had differences in study populations, such as the inclusion of veterans in study Orkaby 2017, (where the proportion of females was low) and the pre-exclusion of patients with contraindications to OAC in study Wang 2023. Excluding these studies with high heterogeneity might affect the overall results. Lastly, based on the funnel plot, the five studies incorporated into the meta-analysis potentially exhibit publication bias. However, due to the limited number of articles included in the consolidated statistics, it may not be appropriate to conduct further quantitative analysis of publication bias (30). Therefore, we believe our study didn't provide solid conclusion, and still should be considered hypothesis-generating. Additional studies are needed to better define the risk-benefit ratio of anticoagulation in patients with dementia.

### 4.5. Prospect of further studies

We recommend that future research should focus on exploring the following areas: (1) Further clarification of the target beneficiary population is warranted. Based on the articles involved in this study, potential subgroups that may experience greater benefits include individuals with improved caregiving conditions, normal kidney function, mild cognitive impairments, and no history of intracranial hemorrhage. (2) A deeper understanding of the advantages of DOACs compared to VKAs within this specific population should be pursued. While conducting randomized controlled trials poses challenges, prospective cohort studies can be undertaken to yield higher-quality clinical evidence.

## 5. Conclusion

Collectively, we found that the administration of OAC in patients with AF and dementia may lead to a reduction in all-cause mortality. The results provide evidence supporting the continued use of OAC in individuals with AF and dementia, thereby enabling more patients to derive benefits from this treatment.

## Data Availability

The original contributions presented in the study are included in the article/**Supplementary Material**, further inquiries can be directed to the corresponding authors.

## References

[1] Wolf PA Abbott RD Kannel WB. Atrial fibrillation as an independent risk factor for stroke: the framingham study. Stroke. (1991) 22(8):983–8. 10.1161/01.str.22.8.983 1866765

[2] January CT Wann LS Calkins H Chen LY Cigarroa JE Cleveland JC Jr. 2019 Aha/Acc/Hrs focused update of the 2014 Aha/Acc/Hrs guideline for the management of patients with atrial fibrillation: a report of the American college of cardiology/American heart association task force on clinical practice guidelines and the heart rhythm society in collaboration with the society of thoracic surgeons. Circulation. (2019) 140(2):e125–51. 10.1161/cir.0000000000000665 30686041

[3] Grymonprez M Capiau A Steurbaut S Mehuys E Boussery K De Backer TL Adherence and persistence to oral anticoagulants in patients with atrial fibrillation: a Belgian nationwide cohort study. Front Cardiovasc Med. (2022) 9:994085. 10.3389/fcvm.2022.994085 36247477 PMC9558210

[4] Jankowska-Polanska B Katarzyna L Lidia A Joanna J Dudek K Izabella U. Cognitive function and adherence to anticoagulation treatment in patients with atrial fibrillation. J Geriatr Cardiol. (2016) 13(7):559–65. 10.11909/j.issn.1671-5411.2016.07.006 27605935 PMC4996829

[5] Seong HJ Lee K Kim BH Son YJ. Cognitive impairment is independently associated with non-adherence to antithrombotic therapy in older patients with atrial fibrillation. Int J Environ Res Public Health. (2019) 16(15):2698. 10.3390/ijerph16152698 31362337 PMC6696263

[6] Dodson JA Petrone A Gagnon DR Tinetti ME Krumholz HM Gaziano JM. Incidence and determinants of traumatic intracranial bleeding among older veterans receiving warfarin for atrial fibrillation. JAMA Cardiol. (2016) 1(1):65–72. 10.1001/jamacardio.2015.0345 27437657 PMC5600874

[7] Subic A Cermakova P Religa D Han S von Euler M Kåreholt I Treatment of atrial fibrillation in patients with dementia: a cohort study from the Swedish dementia registry. J Alzheimers Dis. (2018) 61(3):1119–28. 10.3233/jad-170575 29286925 PMC5798527

[8] Orkaby AR Ozonoff A Reisman JI Miller DR Zhao SB Rose AJ. Continued use of warfarin in veterans with atrial fibrillation after dementia diagnosis. J Am Geriatr Soc. (2017) 65(2):249–56. 10.1111/jgs.14573 28039854

[9] Wang W Lessard D Kiefe CI Goldberg RJ Parish D Helm R Differential effect of anticoagulation according to cognitive function and frailty in older patients with atrial fibrillation. J Am Geriatr Soc. (2023) 71(2):394–403. 10.1111/jgs.18079 36273408 PMC10207283

[10] Wang D Xu X Peng W Wang X Pan G. Clinical Benefits of Oral Anticoagulants in Atrial Fibrillation Patients with Dementia: A Systematic Review and Meta-Analysis: PROSPERO (2023). Available at: https://www.crd.york.ac.uk/prospero/display_record.php?ID=CRD42023420678 10.3389/fcvm.2023.1265331 PMC10507720 37731522

[11] Page MJ McKenzie JE Bossuyt PM Boutron I Hoffmann TC Mulrow CD The prisma 2020 statement: an updated guideline for reporting systematic reviews. BMJ (Clin Res ed). (2021) 372:n71. 10.1136/bmj.n71 PMC8005924 33782057

[12] Stang A. Critical evaluation of the Newcastle-Ottawa Scale for the assessment of the quality of nonrandomized studies in meta-analyses. Eur J Epidemiol. (2010) 25(9):603–5. 10.1007/s10654-010-9491-z 20652370

[13] *Review Manager (Rev.Man.)*. 5.4.1 Version ed. Copenhagen, Denmark: The Cochrane Collaboration (2014).

[14] Madhavan M Holmes DN Piccini JP Ansell JE Fonarow GC Hylek EM Association of frailty and cognitive impairment with benefits of oral anticoagulation in patients with atrial fibrillation. Am Heart J. (2019) 211:77–89. 10.1016/j.ahj.2019.01.005 30901602

[15] Lin KJ Singer DE Bykov K Bessette LG Mastrorilli JM Cervone A Comparative effectiveness and safety of oral anticoagulants by dementia status in older patients with atrial fibrillation. JAMA Netw Open. (2023) 6(3):e234086. 10.1001/jamanetworkopen.2023.4086 36976562 PMC10051113

[16] Fanning L Lau WCY Mongkhon P Man KKC Bell JS Ilomäki J Safety and effectiveness of direct oral anticoagulants vs warfarin in people with atrial fibrillation and dementia. J Am Med Dir Assoc. (2020) 21(8):1058–64.e6. 10.1016/j.jamda.2019.11.022 31917107

[17] Ouellet GM O'Leary JR Leggett CG Skinner J Tinetti ME Cohen AB. Benefits and harms of oral anticoagulants for atrial fibrillation in nursing home residents with advanced dementia. J Am Geriatr Soc. (2023) 71(2):561–8. 10.1111/jgs.18108 36310367 PMC9957933

[18] Cobas Paz R Roubína SR Assia EA Pardalb CB Comesañac JG Lópezd AGC Impact of anticoagulation in patients with dementia and atrial fibrillation. Results of the cardiochuvi-fa registry. Rev Esp Cardio (2020) 73(11):877–84. 10.1016/j.rec.2019.10.025 32081625

[19] Hindricks G Potpara T Dagres N Arbelo E Bax JJ Blomström-Lundqvist C 2020 ESC guidelines for the diagnosis and management of atrial fibrillation developed in collaboration with the European association for cardio-thoracic surgery (EACTS): the task force for the diagnosis and management of atrial fibrillation of the European society of cardiology (ESC) developed with the special contribution of the European heart rhythm association (EHRA) of the ESC. Eur Heart J. (2021) 42(5):373–498. 10.1093/eurheartj/ehaa612 32860505

[20] Buchanan GS Rodgers GM Ware Branch D. The inherited thrombophilias: genetics, epidemiology, and laboratory evaluation. Best Pract Res Clin Obstet Gynaecol. (2003) 17(3):397–411. 10.1016/s1521-6934(03)00010-5 12787534

[21] Bainey KR Welsh RC Connolly SJ Marsden T Bosch J Fox KAA Rivaroxaban plus aspirin versus aspirin alone in patients with prior percutaneous coronary intervention (COMPASS-PCI). Circulation. (2020) 141(14):1141–51. 10.1161/circulationaha.119.044598 32178526

[22] Vargas ER Sposato LA Lee SAW Hachinski V Cipriano LE. Anticoagulation therapy for atrial fibrillation in patients with Alzheimer’s disease. Stroke. (2018) 49(12):2844–50. 10.1161/strokeaha.118.022596 30571418

[23] Grymonprez M Steurbaut S De Backer TL Petrovic M Lahousse L. Effectiveness and safety of oral anticoagulants in older patients with atrial fibrillation: a systematic review and meta-analysis. Front Pharmacol. (2020) 11:583311. 10.3389/fphar.2020.583311 33013422 PMC7509201

[24] Guo Y Kotalczyk A Imberti JF Wang Y Lip GYH. Poor adherence to guideline-directed anticoagulation in elderly Chinese patients with atrial fibrillation: a report from the optimal thromboprophylaxis in elderly Chinese patients with atrial fibrillation (ChiOTEAF) registry. Eur Heart J Qual Care Clin Outcomes. (2023) 9(2):169–76. 10.1093/ehjqcco/qcab054 34370024 PMC9972510

[25] Ruigómez A Vora P Balabanova Y Brobert G Roberts L Fatoba S Discontinuation of non-vitamin K antagonist oral anticoagulants in patients with non-valvular atrial fibrillation: a population-based cohort study using primary care data from the health improvement network in the UK. BMJ Open. (2019) 9(10):e031342. 10.1136/bmjopen-2019-031342 PMC6803078 31630107

[26] Björck F Renlund H Svensson PJ Själander A. Warfarin persistence among stroke patients with atrial fibrillation. Thromb Res. (2015) 136(4):744–8. 10.1016/j.thromres.2015.07.028 26254195

[27] Zhang XL Zhang XW Wang TY Wang HW Chen Z Xu B Off-label under- and overdosing of direct oral anticoagulants in patients with atrial fibrillation: a meta-analysis. Circ Cardiovasc Qual Outcomes. (2021) 14(12):e007971. 10.1161/circoutcomes.121.007971 34932377

[28] Wang W Saczynski JS Lessard D Goldberg RJ Parish D Helm R Presence of geriatric conditions is prognostic of major bleeding in older patients with atrial fibrillation: a cohort study. J Gen Intern Med. (2022) 37(15):3893–9. 10.1007/s11606-022-07410-x 35102482 PMC9640487

[29] Harrison SL Akpan A Lip GYH. Frailty and cognitive impairment are not reasons to withhold anticoagulation in people with atrial fibrillation but screening could guide management. J Am Geriatr Soc. (2021) 69(7):1807–10. 10.1111/jgs.17142 33893638

[30] Egger M Davey Smith G Schneider M Minder C. Bias in meta-analysis detected by a simple, graphical test. BMJ (Clin Res ed). (1997) 315(7109):629–34. 10.1136/bmj.315.7109.629 PMC2127453 9310563

