# Supplementary material for: Clinical benefits of oral anticoagulants in atrial fibrillation patients with dementia: a systematic review and meta-analysis
Source: Front Cardiovasc Med. 2023 Sep 5;10:1265331. doi: 10.3389/fcvm.2023.1265331 (PMC10507720; doi:10.3389/fcvm.2023.1265331)
Supplement: Supplementary file 1 [file Datasheet1.docx]

Supplementary Material

Clinical Benefits of Oral Anticoagulants in Atrial Fibrillation Patients with Dementia: A Systematic Review and Meta-Analysis

**Dayang Wang1,2†, Xiaoqing Xu3†, Xiaowan Han2, Jing Xie2, Hufang Zhou2, Wenhua Peng2* and Guozhong Pan2***

*** Correspondence:** Guozhong Pan: [panguozhong108@sina.com](mailto:panguozhong108@sina.com); Wenhua Peng: [Pengwenhua76@163.com.](mailto:Pengwenhua76@163.com.*)

# Search Strategies

**Pubmed**

((Dementia[Title/Abstract]) OR (Alzheimer[Title/Abstract]) OR (Cognitive impairment[Title/Abstract]) OR (Amentia[Title/Abstract]) OR (Amentia[Title/Abstract]) OR (Vascular Dementia[Title/Abstract]) OR (Lewy Body Disease[Title/Abstract])) AND ((Anticoagulant Drug[Title/Abstract]) OR (Anticoagulant Agents[Title/Abstract]) OR (Anticoagulation[Title/Abstract]) OR (Indirect Thrombin Inhibitors[Title/Abstract]) OR (Antithrombins[Title/Abstract]) OR (Factor Xa Inhibitors[Title/Abstract]) OR (Warfarin[Title/Abstract]) OR (NOAC[Title/Abstract]) OR (Rivaroxaban[Title/Abstract]) OR (Apixaban[Title/Abstract]) OR (Edoxaban[Title/Abstract]) OR (Dabigatran[Title/Abstract]))

**Embase**

((Dementia):ti,ab,kw OR (Cognitive impairment):ti,ab,kw OR (Amentia):ti,ab,kw OR (Vascular Dementia):ti,ab,kw OR (Lewy Body Disease):ti,ab,kw OR (Alzheimer):ti,ab,kw) AND ((Anticoagulant Drug):ti,ab,kw OR (Anticoagulant Agents):ti,ab,kw OR (Anticoagulation):ti,ab,kw OR (Indirect Thrombin Inhibitors):ti,ab,kw OR (Antithrombins):ti,ab,kw OR (Warfarin):ti,ab,kw OR (NOAC):ti,ab,kw OR (Rivaroxaban):ti,ab,kw OR (Apixaban):ti,ab,kw OR (Edoxaban):ti,ab,kw OR (Dabigatran):ti,ab,kw)

**Cochrane Library**

((Dementia):ti,ab,kw OR (Cognitive impairment):ti,ab,kw OR (Amentia):ti,ab,kw OR (Vascular Dementia):ti,ab,kw OR (Lewy Body Disease):ti,ab,kw OR (Alzheimer):ti,ab,kw) AND ((Anticoagulant Drug):ti,ab,kw OR (Anticoagulant Agents):ti,ab,kw OR (Anticoagulation):ti,ab,kw OR (Indirect Thrombin Inhibitors):ti,ab,kw OR (Antithrombins):ti,ab,kw OR (Warfarin):ti,ab,kw OR (NOAC):ti,ab,kw OR (Rivaroxaban):ti,ab,kw OR (Apixaban):ti,ab,kw OR (Edoxaban):ti,ab,kw OR (Dabigatran):ti,ab,kw)

**Web of Science**

#1 TS=(Dementia OR Alzheimer OR Cognitive impairment OR Amentia OR Vascular Dementia OR Lewy Body Disease )

#2 TS=(Anticoagulant Drug OR Anticoagulant Agents OR Anticoagulation OR Indirect Thrombin Inhibitors OR Antithrombins OR Factor Xa Inhibitors OR Warfarin OR NOAC OR Rivaroxaban OR Apixaban OR Edoxaban OR Dabigatran)

#3 #1 AND #2

# Supplementary Figures and Tables

For more information on Supplementary Material and for details on the different file types accepted, please see [here](https://www.frontiersin.org/guidelines/author-guidelines#supplementary-material).

## Supplementary Figures

### Supplementary Figure S1. Sensitive Analysis


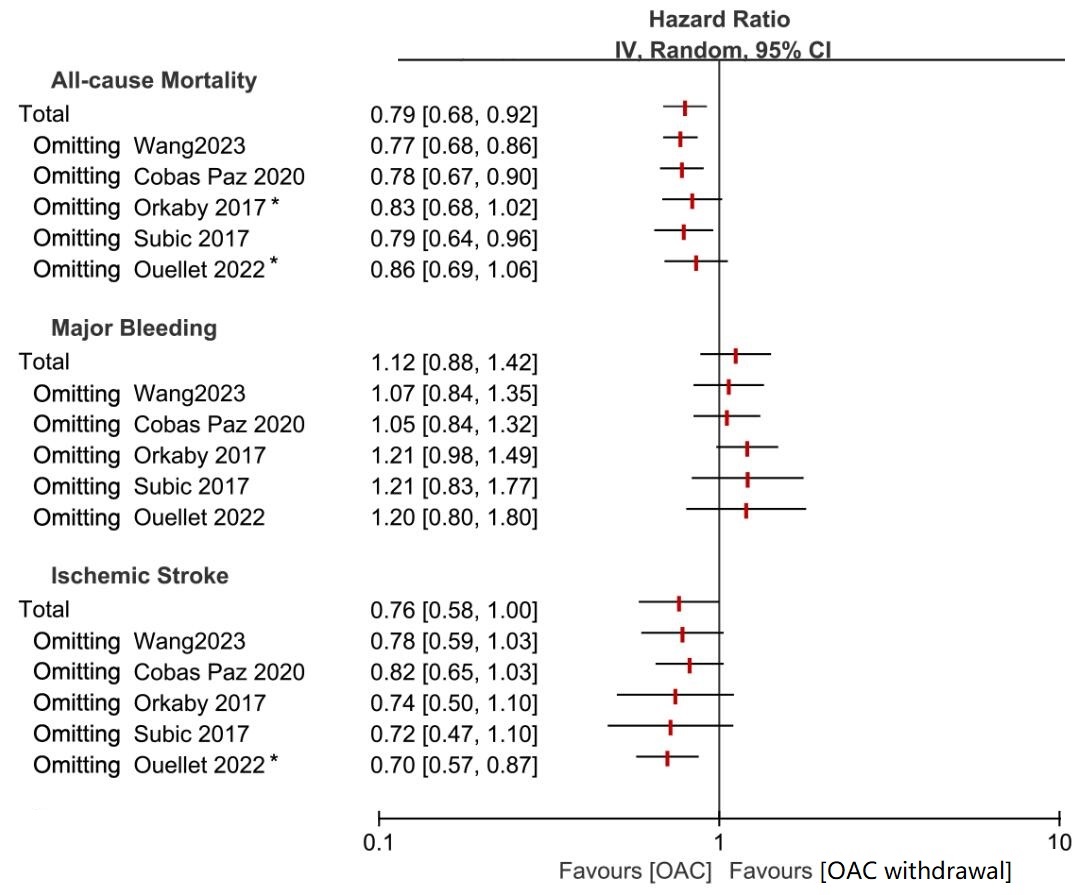


Figure S1. Sensitive analysis of included studies. *Omitting this study may impact the pooled results of the meta-analysis. Abbreviations: CI: confidence interval

### Supplementary Figure S2. Funnel plot of outcomes of OAC vs. non-OAC.


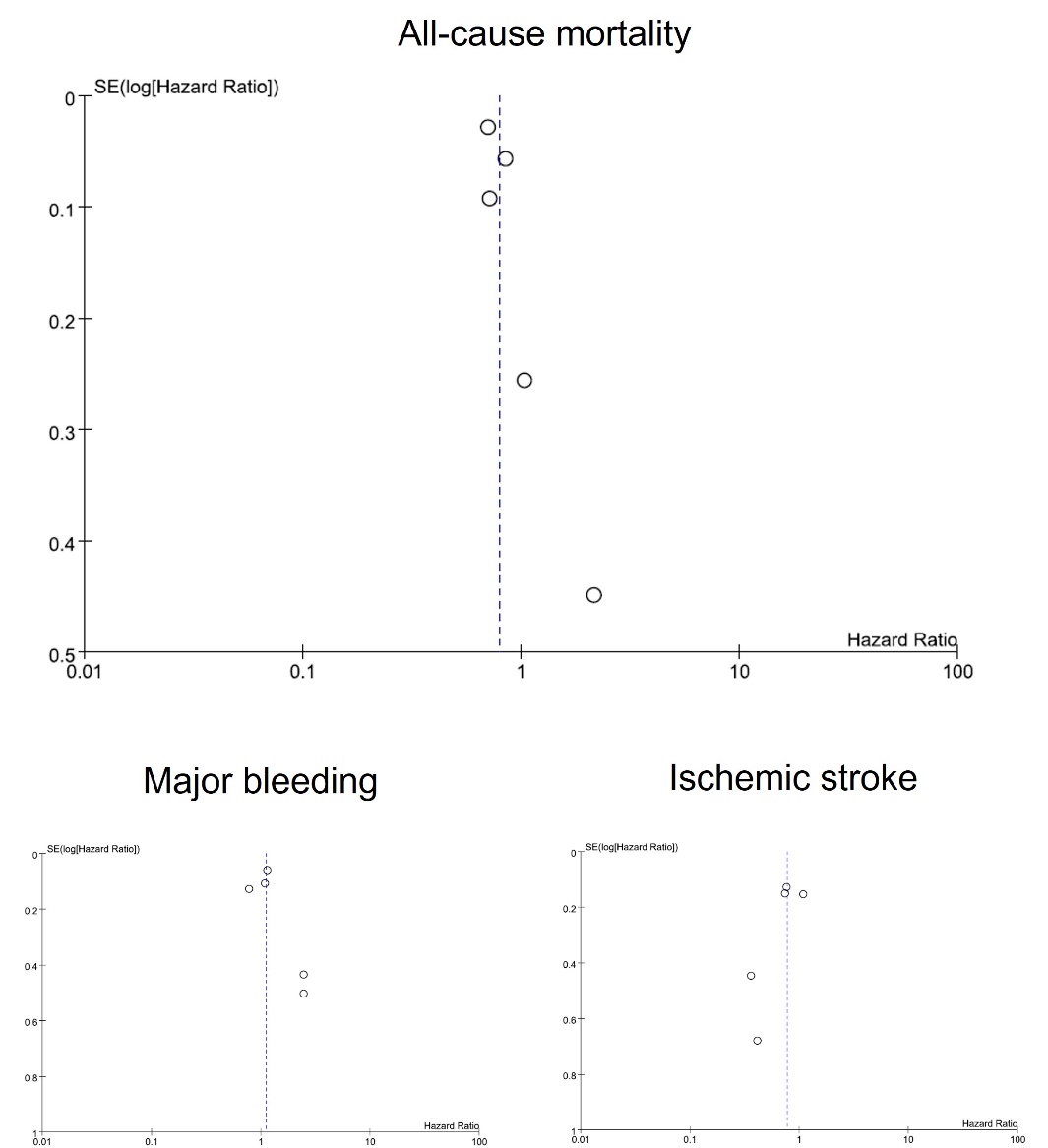


Figure S2. Funnel plot of outcomes of OAC vs. OAC withdrawal in AF patients with dementia.

## Supplementary Tables

**Supplementary Table S1**. Adjusted confounders of the included studies

| Study ID | Adjusted confounders |
| --- | --- |
| Cobas Paz 2020 | age, sex, hypertension, diabetes mellitus, ischemic heart disease, previous stroke or embolism, previous heart failure or left ventricular ejection fraction ≤ 40%, history of bleeding, anemia, CKD-EPI < 60 mL/min/1.73 m2, CHA2DS2-VASc score, HAS-BLED score, antiplatelet therapy, and treatment with beta-blockers, angiotensin-converting enzyme inhibitors or angiotensin receptor blockers, digoxin, statins, and proton pump inhibitors |
| Orkaby 2017 | Age, Race, BMI, EGFR, Coronary artery disease, Congestive heart failure, Stroke, Hypertension, Diabetes mellitus, Peripheral arterial disease, Venous insufficiency, Hyperlipidemia, Cancer diagnosis in the last 6 months, Psychiatric disease, Alcohol abuse, eye disease, Number of medications other than warfarin, CHADS2 score |
| Ouellet 2022 | age, sex, race, medicaid eligible, time in long-term care ≥1 year, heart failure, hypertension, diabetes mellitus, peripheral vascular disease, stroke/transient ischemic attack, myocardial infarction, anemia, chronic kidney disease, bleeding history, lymphoma, solid tumor, metastatic cancer, COPD, liver disease, ischemic heart disease, CHA2DS2VASC SCORE, ATRIA SCORE, antiplatelet use, NSAIDS use, number of total medications, rejection of care, falls, weight loss, pressure ulcer，difficulty swallowing, hospice use, feeding tube |
| Subic 2017 | age, sex, number of medication, MMSE, dementia type (AD versus others), nursing home placement, and previous diagnosis of diabetes, hypertension, heart failure, IS, any-cause hemorrhage, liver diseases, kidney diseases. |
| Wang 2023 | age, race, CHADS-VASC score, HAS-BLED score, Charlson Co-Morbidity Index, type of AF (paroxysmal, persistent, or permanent), fall in the past six months, history of myocardial infarction, history of peripheral vascular disease, history of stroke, history of liver disease, estimated glomerular filtration rate, dual antiplatelet therapy, total medication count, provider type (internist, cardiologist, or electrophysiologist), site |
| Fanning 2020 | age; gender; IS, TIA, or SE; major (ICB or GIB) bleeding; other bleeding (anemia, urinary tract bleeding, respiratory tract bleeding, eye bleeding, and hemopericardium); vascular disease (myocardial infarction or peripheral vascular disease); congestive cardiac failure; moderate to advanced chronic kidney disease; hypertension; diabetes, smoking status; baseline medication use (180 days prior to index date): angiotensin converting enzyme inhibitors, angiotensin receptor blockers, calcium channel blockers, beta-blockers, aspirin or clopidogrel, loop diuretics, nonsteroidal anti-inflammatory drugs, statins, proton pump inhibitors, histamine-2 receptor antagonists, selective serotonin reuptake inhibitors, anti-dementia medications; and CHA2DS2-VASc, HAS-BLED, and Charlson Comorbidity Index scores. |
| Lin 2023 | demographic characteristics, comorbidities, prescription drug use, and health care use. (3 cohorts, 79 covariates in total). |
|  | |
