# Supplementary figures and images for: Clinical benefits of oral anticoagulants in atrial fibrillation patients with dementia: a systematic review and meta-analysis
Source: Front Cardiovasc Med. 2023 Sep 5;10:1265331. doi: 10.3389/fcvm.2023.1265331 (PMC10507720; doi:10.3389/fcvm.2023.1265331)

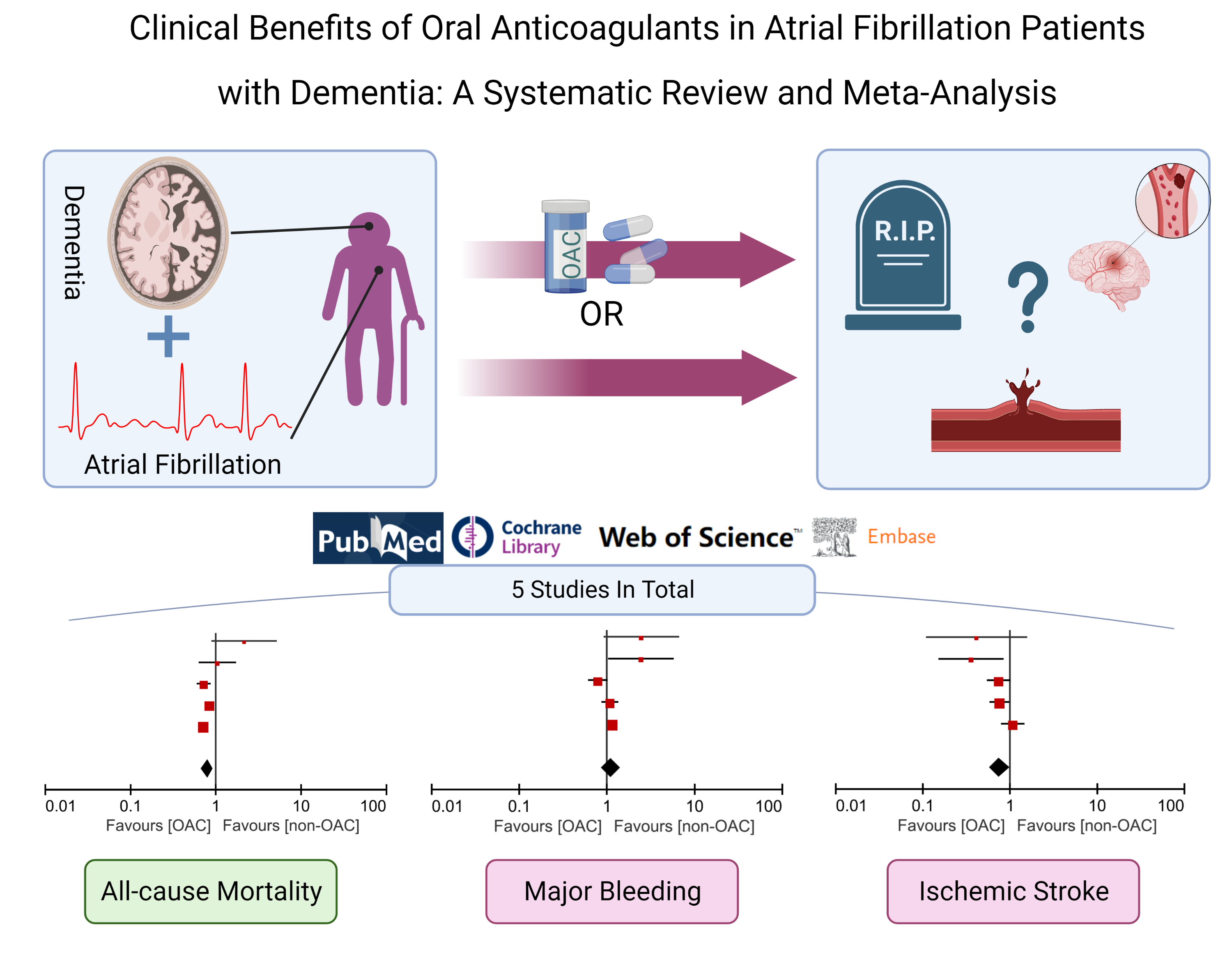

Supplement: Supplementary file 2 [file Image1.jpeg]
